# Association of BAP with urinary albumin excretion in postmenopausal, but not premenopausal, non-CKD Japanese women

**DOI:** 10.1038/s41598-017-18473-w

**Published:** 2018-01-08

**Authors:** Etsuko Ozaki, Shinsuke Yamada, Nagato Kuriyama, Daisuke Matsui, Isao Watanabe, Teruhide Koyama, Yasuo Imanishi, Masaaki Inaba, Yoshiyuki Watanabe

**Affiliations:** 1 Department of Epidemiology for Community Health and Medicine, Kyoto Prefectural University of Medicine Graduate School of Medical Science, 465 Kajii-cho, Kawaramachi-Hirokoji, Kamigyo-ku, Kyoto, 602-8566 Japan; 2 Department of Metabolism, Endocrinology, and Molecular Medicine, Osaka City University Graduate School of Medicine, 1-4-3 Asahi-machi, Abeno-ku, Osaka, 545-8585 Japan

## Abstract

We investigated whether the phosphate (Pi) load in the circulation causes renal damage in non-CKD women. This cross-sectional study included 1,094 non-CKD Japanese women. Fibroblast growth factor (FGF)-23 as a parameter for the Pi load, bone alkaline phosphatase (BAP) as a bone metabolic marker, and the urinary albumin-to-creatinine ratio (UACR) as an early marker for renal damage were measured. Postmenopausal women exhibited significantly higher levels of serum Pi, FGF-23, BAP, and UACR and significantly lower eGFR than premenopausal women. In postmenopausal women, a multiple regression analysis confirmed a correlation between serum BAP and log UACR. In premenopausal women, although serum FGF-23 did not correlate with log UACR, a multiple regression analysis revealed that FGF-23 correlated with log UACR. Based on the i ncrease observed in BAP and its close relationship with log UACR in postmenopausal women, the release of Pi from bone may be linked to the systemic circulation of Pi, which has the potential to induce renal and vascular damage. Therefore, serum FGF-23 may be a useful marker for renal and vascular damage in premenopausal women; however, it currently remains unclear whether FGF-23 by itself or as a surrogate marker for the Pi load induces damage in the kidney and/or vasculature.

## Introduction

It is becoming increasingly apparent that a phosphate (Pi) load in the circulation and resultant hyperphosphatemia exert toxic effects on vascular walls through several mechanisms^1^, even in subjects without chronic kidney disease (CKD)0^2^. Serum fibroblast growth factor (FGF)-23 levels increase in response to a Pi overload in order to protect against the development of hyperphosphatemia^3^. A previous study demonstrated that the rate of entry of Pi into the circulation was influenced by its absorption at the intestines and release from bone, while the rate of Pi outflow was only assessed by its urinary excretion at the kidneys^4^. Since we recently reported that serum FGF-23 acts physiologically to regulate serum Pi levels from the standpoint of vitamin D metabolism in Japanese non-CKD subjects with eGFR ≥ 60 mL/min/1.73 m^2^ ^5^, serum FGF-23 has potential as a clinically useful surrogate marker of the Pi load in the circulation in non-CKD subjects. In female subjects, bone resorption abruptly increases at the time of menopause due to the loss of bone-protective estrogen, which markedly increases the release of Pi from bone as a result of stimulated bone resorption, based on our finding that the administration of denosumab to Japanese patients with postmenopausal osteoporosis suppressed serum Pi levels by approximately 20% 8 days after its administration (personal observation). Therefore, postmenopausal women differ from premenopausal women in that the source of Pi, which mainly enters the circulation, may be bone, and this may be evaluated by serum bone alkaline phosphatase (BAP), a reliable bone metabolic marker of osteogenesis^6^.

There is increasing evidence for the toxic effects of increased serum Pi and/or FGF-23 on the kidneys in CKD patients^7^. The urine albumin-to-creatinine ratio (UACR) is recognized as the earliest and most precise marker of kidney damage and cardiovascular mortality in CKD patients^8^.

The present study aimed to clarify whether serum Pi, FGF-23, and BAP are independently associated with UACR in non-CKD women, and investigate differences in the origin of the Pi load between pre- and postmenopausal non-CKD women in an attempt to elucidate the relationships among serum FGF-23, BAP, and UACR.

## Subjects and Methods

### Participants

**Figure 1:**
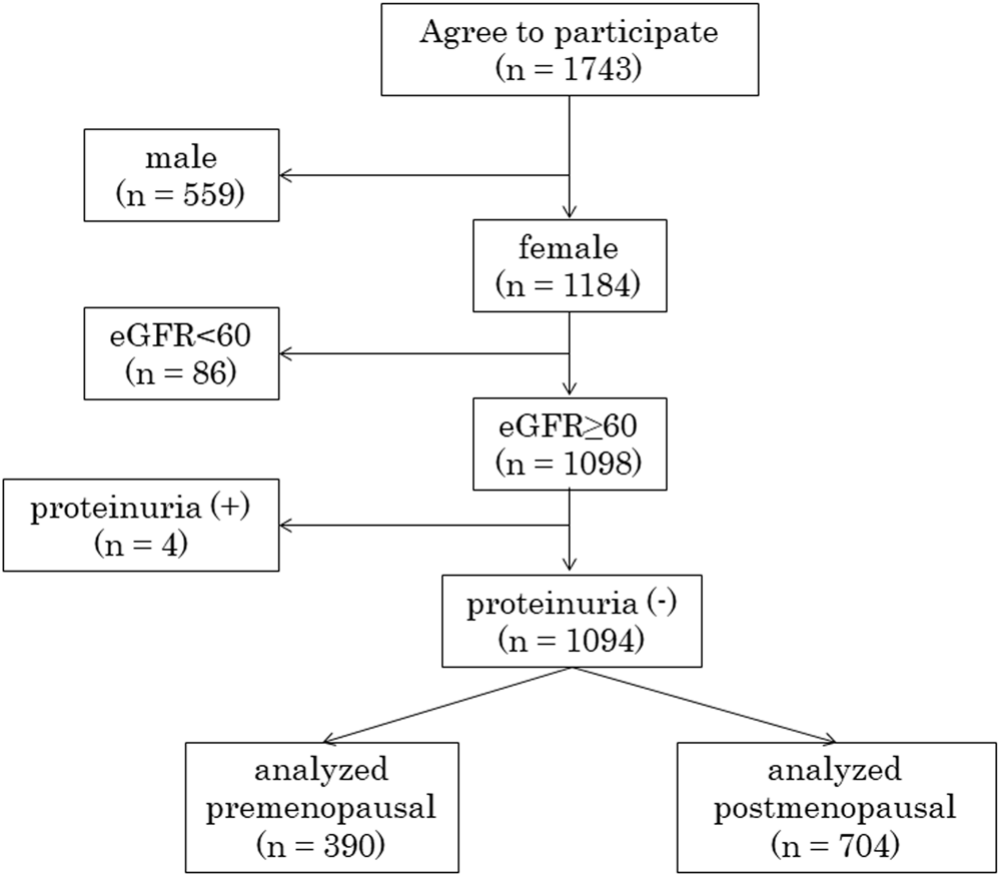
Flowchart of this study design.

As part of the Japan Multi-Institutional Collaborative Cohort (J-MICC) Study, a large cohort study initiated in 2005 to elucidate the genetic-environmental interactions of lifestyle-related diseases including cancer in Japan, we conducted a baseline survey on approximately 6,500 inhabitants older than 35 years of age in Kyoto prefecture between 2007 and 2013 (the J-MICC Study Kyoto field)^9^. Subjects were restricted to females, and were further excluded based on an eGFR < 60 mL/min or a dipstick testing positive for proteinuria. Subjects who had been continuously taking medication such as that affecting bone and calcium/Pi metabolism were also excluded from the present study. Therefore, 1,094 female non-CKD subjects (390 premenopausal women and 704 postmenopausal women) were enrolled in the present cross-sectional study (Fig. 1). Among these subjects, 140 (12.8%) had hypertension, 153 (14.0%) hyperlipidemia, and 23 (2.1%) diabetes (Table 1). The criteria for diagnoses of hypertension, hyperlipidemia, and diabetes in the present study were systolic/diastolic blood pressure ≥ 140/90 mmHg, LDL-C ≥ 140 mg/dL, HDL-C ≤ 40 mg/dL, and/or triglycerides ≥ 150 mg/dL, and A1C ≥ 6.5%, respectively, or receiving current treatments for each disease. Although we did not confirm the types of drugs administered, the numbers of subjects that received antihypertensive, antidiabetic, and dyslipidemic agents were 4 (1.0%), 0 (0%), and 4 (1.0%), respectively, among 390 premenopausal women, and 100 (14.2%), 16 (2.3%), and 119 (16.9%), respectively, among 704 postmenopausal women. All participants provided written informed consent before participating in this study, which was approved by the Institutional Ethics Committee at Kyoto Prefectural University of Medicine (RBMR-E-289), and was conducted in accordance with the principals of the Declaration of Helsinki.

**Table 1:** Clinical and biochemical profiles of non-CKD female subjects

Measures	All	Menses		p value
		Pre menopausal	Post menopausal	
n (%)	1094	390 (36)	704 (64)	—
Age (years)	53.8 (10.1)	43.2 (4.9)	59.7 (7.0)	<0.001
BMI (kg/m^2^)	21.8 (3.2)	21.7 (3.3)	21.9 (3.1)	0.024
SBP (mmHg)	130.3 (21.4)	121.6 (17.9)	135.1 (21.6)	<0.001
DBP (mmHg)	77.0 (11.5)	74.6 (10.9)	78.3 (11.6)	<0.001
Protein intake (g/day)	66.3 (18.3)	63.9 (17.7)	67.7 (18.6)	0.001
Drinking (n, %)	539 (49.3)	210 (53.8)	329 (46.7)	0.024
Smoking (n, %)	63 (5.8)	27 (6.9)	36 (5.1)	0.219
Hypertension (n, %)	140 (12.8)	13 (3.3)	127 (18.0)	<0.001
Hyperlipidemia (n, %)	153 (14.0)	14 (3.6)	139 (19.7)	<0.001
Diabetes (%)	23 (2.1)	2 (0.5)	21 (3.0)	0.006
Users of medication for hypertension (n, %)	104 (9.5)	4 (1.0)	100 (14.2)	<0.001
Users of medication for diabetes (n, %)	16 (1.5)	0 (0)	16 (2.3)	<0.001
Users of medication for hyperlipidemia (n, %)	123 (11.2)	4 (1.0)	119 (16.9)	<0.001
eGFR (mL/min/1.73 m^2^)	81.7 (12.8)	87.4 (13.4)	78.5 (11.4)	<0.001
Ht (%)	40.4 (3.0)	39.3 (3.4)	41.0 (2.6)	<0.001
Alb (g/dL)	4.5 (0.2)	4.5 (0.2)	4.5 (0.2)	0.117
Ca (mg/dL)	9.3 (0.4)	9.2 (0.3)	9.4 (0.5)	<0.001
P (mg/dL)	3.7 (0.5)	3.7 (0.5)	3.8 (0.4)	<0.001
HbA1C (%)	5.4 (0.4)	5.2 (0.3)	5.5 (0.4)	<0.001
TC (mg/dL)	218.4 (35.7)	201.9 (31.3)	227.6 (34.7)	<0.001
HDL-C (mg/dL)	78.5 (18.8)	78.3 (17.6)	78.7 (19.4)	0.784
LDL-C (mg/dL)	124.7 (31.4)	113.1 (28.1)	131.1 (31.3)	<0.001
TG (mg/dL)	111.4 (69.4)	91.9 (53.1)	122.2 (74.9)	<0.001
FGF-23 (pg/mL)	24.9 (7.0)	23.1 (6.0)	25.9 (7.3)	<0.001
BAP (U/L)	12.0 (4.7)	9.2 (3.0)	13.6 (4.7)	<0.001
UACR (mg/g Cr)	5.84 [4.18-8.65]	5.05 [3.88–7.33]	6.30 [4.50–9.48]	<0.001

Continuous variables are summarized as the mean (SD), whereas medians (interquartile range) are shown for variables with a skewed distribution. Prevalence was reported as a percentage. Subjects were divided into 2 groups based on the presence of a menstrual cycle and then compared. Unpaired samples were analyzed by Mann-Whitney U tests, whereas categorical data were analyzed by χ^2^ tests.

### Measurements

All routine laboratory measurements were performed using standard assays with automated methods^10–12^. Blood samples, obtained at the same time as the baseline survey of the J-MICC Study Kyoto field, were stored at −80 °C until assayed. Urine was collected from the second urination in the morning of the survey day. eGFR was calculated using the new Japanese coefficient for the abbreviated Modification of Diet in Renal Disease Study equation, including a correction factor of 0.739 for women^13^. Urinary albumin and creatinine were measured simultaneously, from which UACR was calculated and expressed in milligrams per gram of creatinine^14^.

Serum FGF-23 was measured using a fully automated random access chemiluminescence immunoanalyzer, the CL-JACK^®^ System (Kyowa Medex Co. Ltd., Tokyo, Japan; intra-assay coefficient of variation (CV) of 2.7%–3.4%^15^, and inter-assay CV of 1.9%–6.3% (internal data)). Serum BAP was measured with an enzyme immunoassay (ALKPHASE-B; Metra Biosystems)^12^. The intra-assay CV for BAP was 2.2%^10,12^.

### Statistical analysis

Continuous variables with a normal distribution were expressed as the mean ± SD. The median [interquartile range] was used for UACR because of its skewed distribution. Differences between the means of unpaired samples were parametrically analyzed using the Mann-Whitney U test for continuous variables, and Pearson’s chi-squared test for categorical variables. Correlation coefficients were calculated by simple and multiple regression analyses after the logarithmic transformation of UACR because of its transformation to an approximated normal distribution. A univariate regression analysis was performed using Spearman’s rank correlation analysis. Variables were analyzed using a multivariate logistic regression to identify independent predictors of UACR. A *p* value of less than 0.05 was considered to be significant. Results were expressed as odds ratios (OR) and 95% confidence intervals (95% CI). All statistical analyses were performed with SPSS statistics 22 (SPSS Inc. USA).

## Results

### Clinical characteristics of enrolled non-CKD subjects

**Figure 2:**
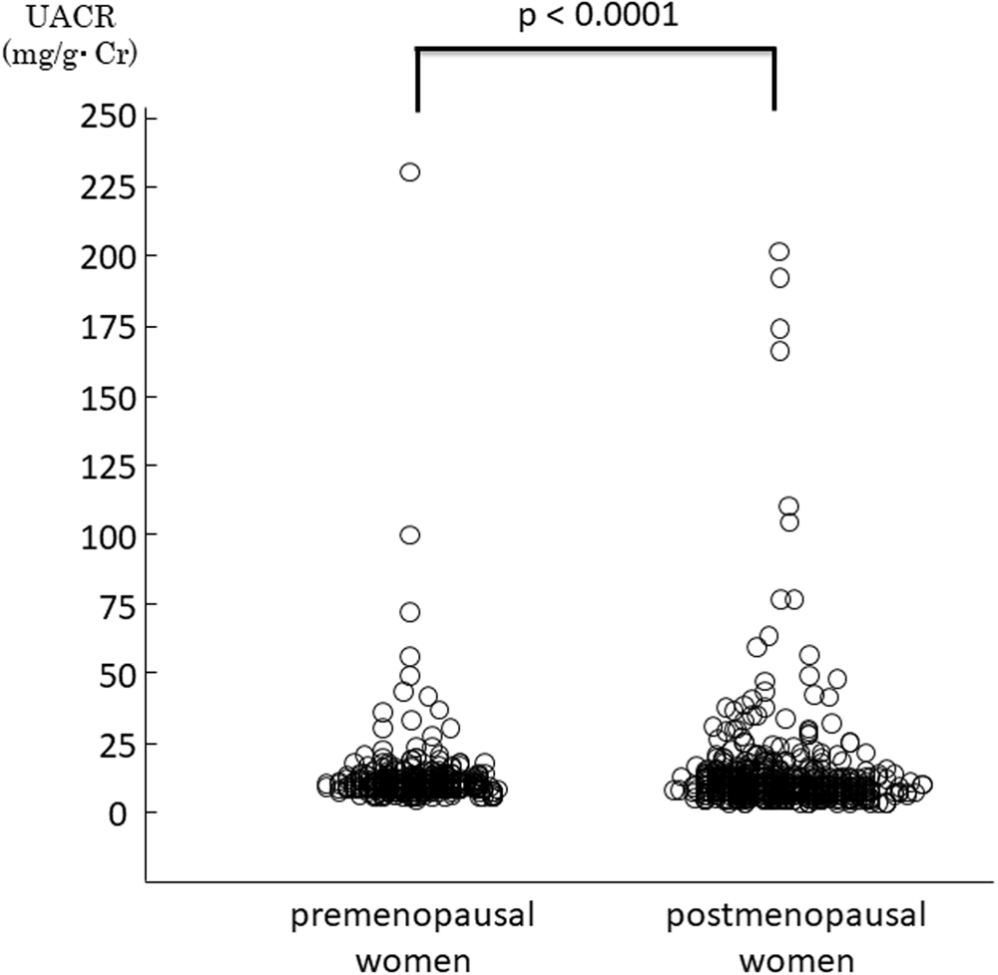
UACR levels were significantly higher in postmenopausal women than in premenopausal women (p < 0.001).

**Figure 3:**
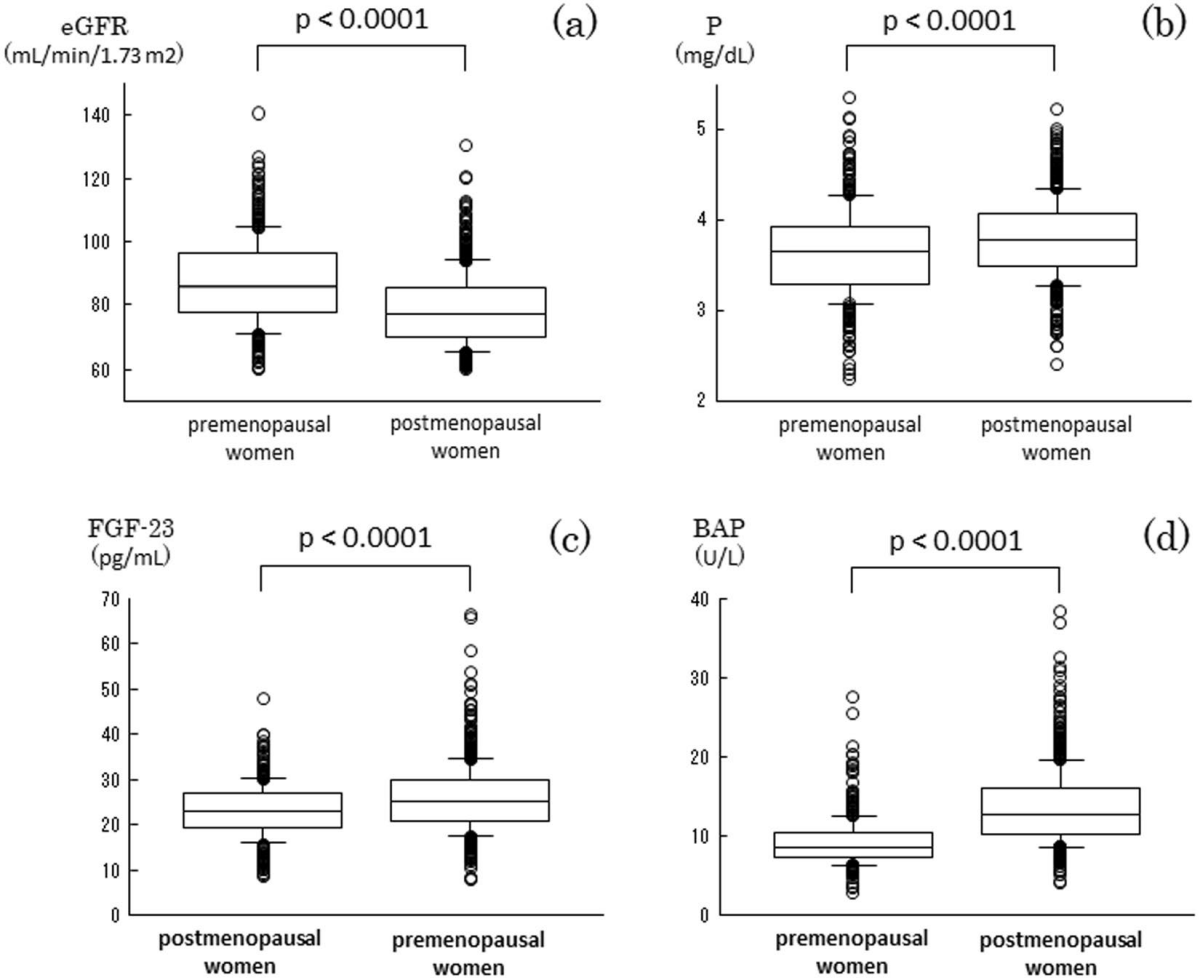
Serum levels of Pi, FGF-23, and BAP were significantly higher, while those of eGFR were significantly lower in postmenopausal women than in premenopausal women (all p < 0.001).

The clinical characteristics of 390 premenopausal and 704 postmenopausal women enrolled in this study are shown in Table 1. The mean ± SD value of eGFR in postmenopausal women was 78.5 ± 11.4 mL/min/1.73 m^2^, which was significantly lower than that in premenopausal women (87.4 ± 13.4 mL/min/1.73 m^2^). Serum Pi, FGF-23, BAP, and UACR were all significantly higher in postmenopausal women than in premenopausal women (Figs 2, 3). Although the absolute difference in serum Pi between pre- and postmenopausal women was not sufficiently large to remain in the normal range, we observed significant differences between the 2 groups (p < 0.001).

### Relationships among eGFR, serum Pi, and serum FGF-23 in postmenopausal, but not premenopausal women

**Figure 4:**
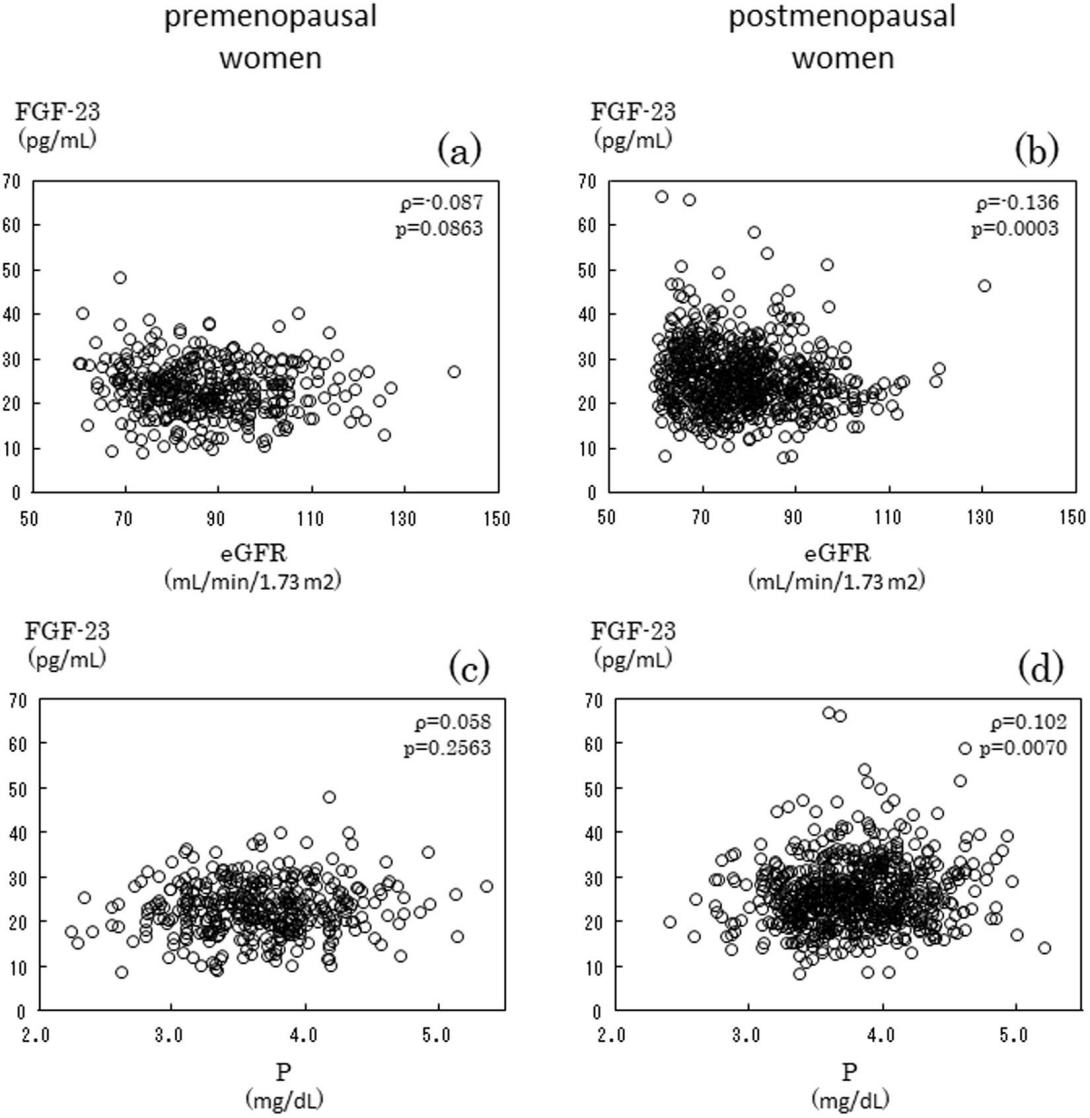
Serum FGF-23 negatively correlated with eGFR in postmenopausal women (**b**), but not in premenopausal women (**a**). Serum FGF-23 positively correlated with serum Pi in postmenopausal women (**d**), but not in premenopausal women (**c**).

Although the present study restricted enrollment to female subjects with eGFR ≥ 60 mL/min/1.73 m^2^, eGFR still negatively correlated with serum FGF-23 in postmenopausal women (ρ = −0.136, p < 0.001) (Fig. 4b), but not premenopausal women (ρ = −0.087, p = 0.086) (Fig. 4b). Furthermore, a positive correlation was observed between serum Pi and FGF-23 in postmenopausal women (ρ = 0.102, p = 0.007) (Fig. 4d), but not premenopausal women (ρ = 0.058, p = 0.257) (Fig. 4c).

### Relationship between serum BAP and log (UACR) in postmenopausal, but not premenopausal women

**Figure 5:**
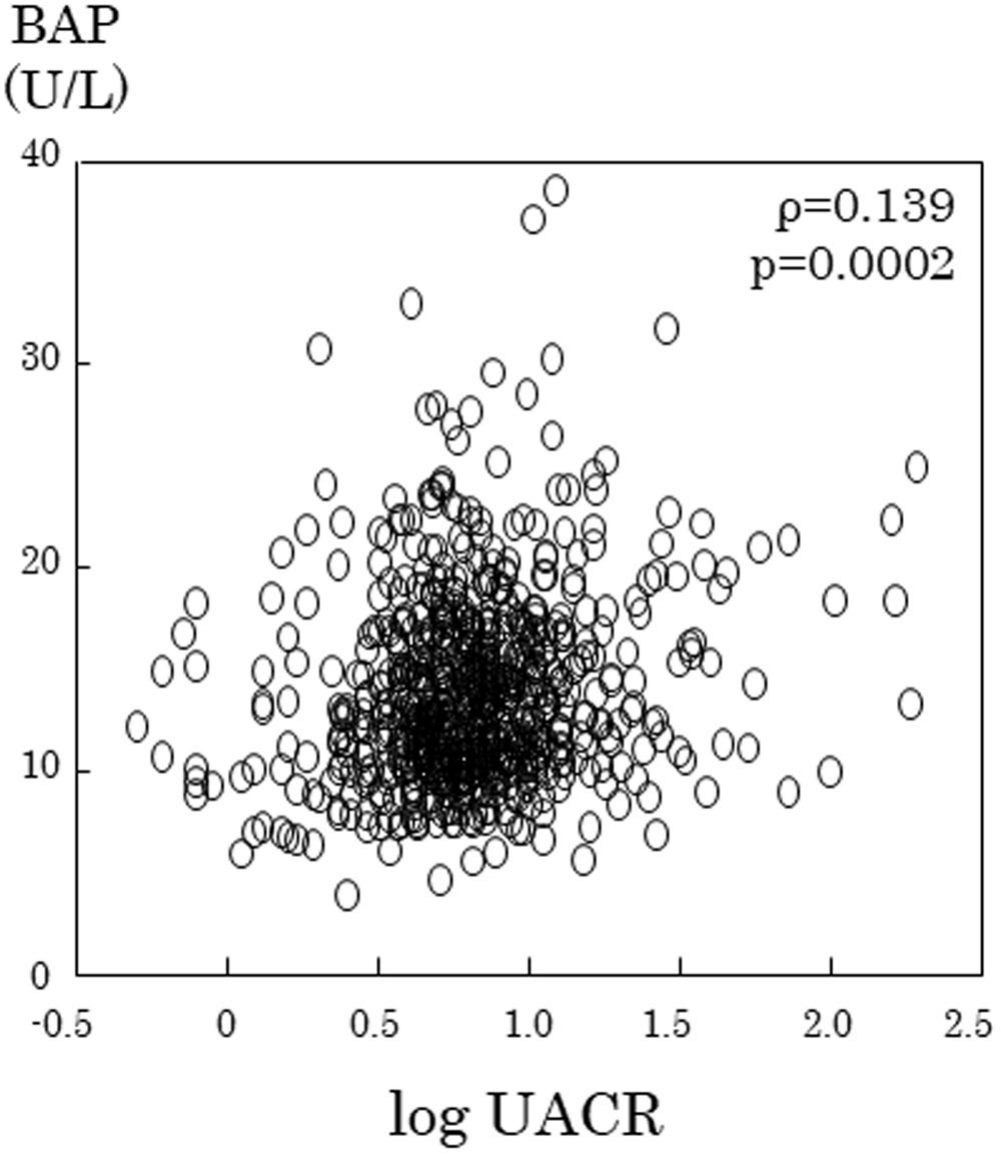
Serum BAP positively correlated with log UACR in postmenopausal women.

The release of Pi from bone into the circulation is assumed to be enhanced in postmenopausal women due to increased bone resorption because of an estrogen deficiency, as reflected by significantly higher serum BAP in postmenopausal women than in premenopausal women (Fig. 3). Therefore, the larger Pi load from bone may result in higher serum FGF-23 and Pi in postmenopausal women than in premenopausal women. Since an increased Pi load is known to increase urinary protein excretion, we examined the relationships among serum FGF-23, Pi, BAP, which are parameters for the Pi load in the circulation, and log (UACR) separately in pre- and postmenopausal women (Table 2). Serum BAP positively correlated with log (UACR) in postmenopausal women (Fig. 5), but not in premenopausal women. Neither serum Pi nor FGF-23 correlated with log (UACR) in pre- or postmenopausal women.

**Table 2:** Univariate correlations between clinical variables and log UACR in non-CKD women.

measures	log UACR			
	Premenopausal women		Postmenopausal women	
	ρ	p	ρ	p
Age	0.068	0.183	0.197	<0.001
BMI	0.008	0.873	0.086	0.023
SBP	0.040	0.426	0.193	<0.001
protein intake	−0.118	0.020	−0.001	0.989
eGFR	0.112	0.028	0.126	0.001
Alb	−0.042	0.403	−0.062	0.102
Serum Ca	−0.060	0.240	−0.021	0.581
Serum P	0.043	0.399	−0.025	0.512
HbA1C	0.056	0.269	0.039	0.303
LDL-C	0.071	0.160	0.047	0.212
HDL-C	−0.085	0.094	−0.108	0.004
TG	0.077	0.129	0.032	0.389
BAP	−0.016	0.757	0.139	<0.001
FGF-23	0.059	0.249	−0.016	0.675

Overall p values were assessed by Spearman’s rank correlation analysis.

### Multivariate analyses to elucidate factors associated with log (UACR) in pre- and postmenopausal women

In the multivariate model that included age, BMI, systolic blood pressure, protein intake, eGFR, serum albumin, serum Ca, serum Pi, HbA1C, HDL-cholesterol, LDL-cholesterol, triglycerides, serum BAP, and serum FGF-23, in addition to the use of medication to treat hypertension, diabetes, and hyperlipidemia in postmenopausal women only, as independent variables, serum BAP was identified as a significant factor in postmenopausal, but not premenopausal women (Tables 3 and 4). Although serum FGF-23 did not correlate with log (UACR) in a simple regression analysis in pre- or postmenopausal women (Table 2), it was identified as a significant and independent factor that positively associated with log UACR in premenopausal, but not postmenopausal women (Tables 3 and 4).

**Table 3:** Multiple regression analyses associated with log UACR in non-CKD postmenopausal women.

covariates	log UACR					
	Model 1		Model 2		Model 3	
	β	p	β	p	β	p
Age	0.122	0.004	0.137	0.001	0.121	0.004
BMI	0.037	0.378	0.047	0.264	0.040	0.342
SBP	0.146	<0.001	0.152	<0.001	0.145	<0.001
protein intake	−0.004	0.905	−0.007	0.853	−0.004	0.917
eGFR	0.145	<0.001	0.146	<0.001	0.143	<0.001
Alb	−0.101	0.015	−0.096	0.021	−0.101	0.015
Serum Ca	0.054	0.202	0.063	0.133	0.054	0.200
Serum P	0.004	0.925	−0.001	0.989	0.006	0.883
HbA1C	−0.082	0.068	0.077	0.085	−0.081	0.071
LDL-C	0.031	0.447	0.033	0.415	0.030	0.460
HDL-C	−0.053	0.227	−0.060	0.177	−0.053	0.227
TG	−0.037	0.376	−0.039	0.355	−0.036	0.398
Medication for hypertension	0.168	<0.001	0.171	<0.001	0.169	<0.001
Medication for diabetes	0.011	0.792	0.013	0.767	0.012	0.771
Medication for hyperlipidemia	−0.038	0.345	−0.047	0.247	−0.039	0.332
BAP	0.089	0.018			0.088	0.019
FGF23			−0.019	0.610	−0.017	0.651
R2 (p)	0.100 (<0.001)		0.093 (<0.001)		0.099 (<0.001)	

β is the standardized regression coefficient.

**Table 4:** Multiple regression analysis associated with log UACR in non-CKD premenopausal women.

covariates	log UACR					
	Model 1		Model 2		Model 3	
	β	p	β	p	β	p
Age	0.072	0.224	0.084	0.155	0.079	0.182
BMI	−0.027	0.660	−0.047	0.444	−0.042	0.498
SBP	0.061	0.299	0.050	0.396	0.053	0.359
protein intake	−0.105	0.040	−0.105	0.039	−0.108	0.035
eGFR	0.095	0.075	0.106	0.048	0.105	0.048
Alb	−0.050	0.430	−0.058	0.357	−0.057	0.364
Serum Ca	−0.052	0.394	−0.057	0.350	−0.056	0.357
Serum P	0.073	0.181	0.060	0.272	0.062	0.259
HbA1C	0.045	0.394	0.043	0.412	0.043	0.412
LDL-C	0.027	0.639	0.042	0.471	0.032	0.582
HDL-C	−0.054	0.362	−0.054	0.364	−0.060	0.310
TG	0.023	0.689	0.018	0.750	0.023	0.688
BAP	−0.070	0.177			−0.074	0.150
FGF23			0.102	0.049	0.105	0.042
R2 (p)	0.018 (0.099)		0.023 (0.056)		0.026 (0.046)	

β is the standardized regression coefficient.

### Multivariable logistic regression analysis of BAP and FGF-23 for UACR in pre- and postmenopausal women

The upper limit values of normal ranges in UAE, BAP, and FGF-23 are 30 mg/dL, 20 U/L, and 50 pg/mL, respectively. The numbers of subjects with higher than the normal level of each parameter were 9 (2.3%), 0 (0%), and 5 (1.3%), respectively, among 390 premenopausal women, and 26 (3.7%), 65 (9.2%), and 6 (0.9%), respectively, among 704 postmenopausal women in the present study. Therefore, we divided all subjects into two groups according to median UAE (5.05 mg/g Cr in premenopausal women and 6.30 mg/g Cr in postmenopausal women) as well as approximate median BAP (10 U/L) and FGF-23 (25 pg/mL), and analyzed UAE as a categorical variable in a logistic regression analysis (Table 5). The results obtained showed that higher BAP levels in postmenopausal women (OR = 1.492; 95% CI = 1.001–2.224; p = 0.049) and higher FGF-23 levels in premenopausal women (OR = 1.679; 95% CI = 1.078–2.613; p = 0.022) were positively associated with higher UACR levels, but not lower BAP or FGF-23 levels.

**Table 5:** Multivariable logistic regression analysis of Pi load factors for UACR in pre- and postmenopausal women.

variables	Premenopausal women*				Postmenopausal women**			
	OR	95% CI		p	OR	95% CI		p
		lower	Upper			lower	Upper	
BAP (U/L)								
<10	1.000	—	—	—	1.000	—	—	—
≥10	1.101	0.696	1.743	0.680	1.492	1.001	2.224	0.049
FGF-23 (pg/mL)								
<25	1.000	—	—	—	1.000	—	—	—
≥25	1.679	1.078	2.613	0.022	0.912	0.655	1.270	0.584

OR odds ratio, Cl confidence interval.

All subjects were divided into two groups according to median UAE (5.05 mg/g Cr in premenopausal women and 6.30 mg/g Cr in postmenopausal women) as well as approximate median BAP (10U/L) and FGF-23 (25 pg/mL).

*Adjusted for age, BMI, SBP, protein intake, eGFR, Alb, serum Ca, serum P, HbA1C, LDL-C, HDL-C, and TG.

**Adjusted for age, BMI, SBP, protein intake, eGFR, Alb, serum Ca, serum P, HbA1C, LDL-C, HDL-C, TG, and the use of medication for hypertension, hyperlipidemia, and diabetes.

## Discussion

The present study demonstrated that (i) postmenopausal women had significantly higher levels of serum Pi, FGF-23, BAP, and UACR and significantly lower eGFR than premenopausal women, and that (ii) serum FGF-23 negatively and positively correlated with eGFR and serum Pi, respectively, in postmenopausal women with eGFR ≥ 60 mL/min/1.73 m^2^, in contrast to premenopausal women. Furthermore, a positive correlation was detected between serum BAP and log UACR in postmenopausal, but not premenopausal women, and the independent correlation between serum BAP and log UACR was confirmed by a multivariate regression analysis.

The amount of the Pi load in the circulation may be influenced by three limiting steps including Pi absorption at the intestines, Pi release from bone due to bone resorption, and Pi excretion into urine at the kidneys^4^. Since postmenopausal women exhibited significantly higher serum BAP and lower eGFR, they are assumed to have a larger Pi load in the circulation due to its greater release from bone simultaneously with its lower excretion into urine than premenopausal women. Therefore, the larger Pi load is assumed to increase serum Pi to a significantly higher level in postmenopausal women than in premenopausal women. Serum FGF-23 positively correlated with serum Pi in postmenopausal women, in contrast to premenopausal women. Serum FGF-23 is now established as a clinically reliable surrogate marker for the Pi load in the circulation because of the increase observed in serum FGF-23 in response to the Pi load^3,16^. Since bone resorption becomes prominent in postmenopausal women due to estrogen deficiency-induced postmenopausal osteoporosis^17^, the amount of Pi released from bone needs to be increased based on the findings of an approximately 17% reduction in serum Pi 7 days after the administration of denosumab to postmenopausal women (unpublished data). Furthermore, we previously reported that cinacalcet, a calcium receptor agonist that inhibits the secretion of PTH from the parathyroid gland, significantly suppressed serum Pi in hemodialysis patients, and this correlated with decreases in serum PTH, either intact or whole PTH, and serum tartrate-resistant acid phosphatase (TRACP)-5b, a bone resorption marker^18^. Taken together with the significantly lower eGFR in postmenopausal women, the larger Pi load in postmenopausal women, either from its enhanced release from bone or impaired renal excretion, may lead to a significant relationship between serum FGF-23 and Pi (Fig. 4).

Serum Pi levels have been established as one of the major biological toxins to blood vessels^1,19^ that increase cardiovascular mortality, particularly in CKD patients, in the international DOPPS cohort study^20^. A previous study reported that reductions in serum Pi and urinary Pi excretion significantly reduced urinary protein excretion in patients with nephrotic syndrome^21^. Furthermore, higher serum Pi, even within the normal range, has recently been suggested to function as a relevant predictor for the development of microalbuminuria^7^ and CVD^2^ in a general population without evidence of CKD, supporting the notion that a slight increase in the Pi load in the circulation may exert harmful effects in the kidneys and blood vessels, even under non-CKD conditions. Therefore, we investigated the relationship between markers for the Pi load in serum (serum Pi, BAP, and FGF-23) and urinary albumin excretion expressed as UACR, a clinically reliable marker for kidney damage. Serum BAP, but not serum Pi or FGF-23, positively correlated with log UACR (ρ = −0.139, p < 0.001) in postmenopausal, but not premenopausal women. Since serum BAP is a representative bone metabolism-related marker, increased levels of BAP may reflect, directly or indirectly, increases in serum Pi from human bone tissue. A multiple regression analysis identified serum BAP, but not serum Pi or FGF-23, as a significant factor positively associated with log UACR in postmenopausal women (Table 3), suggesting that the larger Pi load increasing UACR in postmenopausal women mainly originated from bone as a result of enhanced bone resorption.

Due to the predominance of Pi in bone tissue in the form of hydroxyapatite, we previously reported that the inhibition of bone resorption by the treatment with cinacalcet, which suppresses PTH secretion, correlated with reductions in serum Pi in hemodialysis patients with secondary hyperparathyroidism^18^. Furthermore, a selective estrogen receptor modulator, a direct bone anti-resorptive agent, significantly attenuated age-related decreases in eGFR in osteoporotic females^22^, while estrogen derivatives controlled the increase in serum PTH with compromised renal function due to aging^23^. Furthermore, we previously demonstrated that the administration of risedronate for 1 year significantly suppressed the progression of arterial wall thickening and stiffening, as estimated by the intima-medial thickness of the common carotid artery and pulse-wave velocity, respectively, in postmenopausal osteoporotic patients^24^. In support of the hypothesis that a treatment to suppress bone resorption in patients with uremic hyperparathyroidism or postmenopausal osteoporosis protects against atherosclerotic changes, a recent study demonstrated that the administration of bisphosphonate significantly protected against the development of acute myocardial infarction in osteoporotic patients^25^. Baseline levels of serum Pi were subsequently identified as a significant factor affecting the rate of decline in eGFR^26^. Taken together with the significant suppression of serum Pi by approximately 10% following an injection of denosumab, these findings suggest that bone resorption accelerates the age-related progression of atherosclerotic changes and renal dysfunction by increasing the release of Pi from bone into the circulation. Therefore, the mechanism by which serum BAP correlates with log UACR in postmenopausal subjects may involve the larger amount of Pi released from bone into the circulation.

Although serum FGF-23 did not correlate with log UACR in pre- or postmenopausal women, a multiple regression analysis revealed that serum FGF-23 independently correlated with log UACR in premenopausal, but not postmenopausal women (Table 4). Therefore, elevated serum FGF-23 by itself or as a surrogate marker for a larger Pi load may increase log UACR in premenopausal women, which are both hypothesized to be a definite CVD risk in the general population^2^. The precise influence of FGF 23 on the Pi load currently remains unclear; the reason for the relationship between serum FGF-23 and log UACR in premenopausal women only may be that serum FGF-23 increased from an early stage, reducing the influence of the Pi load due to a slight reduction in renal function. In contrast, the Pi load in postmenopausal women was too large to increase serum Pi in spite of greater increases in serum FGF-23.

The present results demonstrated that postmenopausal women were more obese and had significantly higher TC, LDL-C, and TG levels than premenopausal women, whereas HDH levels did not significantly differ between these groups (Table 1). Previous studies reported that HDL-C was affected markedly less by aging^27^ and menopause^28^ than TC, LDL-C, and TG. These findings support our result showing that only HDL-C levels did not significantly differ between pre- and postmenopausal women.

GFR was positively and protein intake was negatively associated with UACR, as shown in Fig. 4, whereas a high protein intake is generally considered to decrease GFR and UACR. Previous studies showed that BMI negatively correlated with UACR in healthy Japanese high school boys^29^ and high UACR values had a higher waist-hip ratio among women (mean age 37.9 yr) in a Chinese community^30^. These findings indicate that UACR increases with a low muscle quantity in healthy subjects. Although eGFR and protein intake may reflect muscle quantity in healthy and younger subjects, we did not evaluate muscle quantity in the present study; therefore, further studies to clarify this relationship are warranted.

The present study has several limitations. Since relationships among clinical variables were examined within narrow ranges, the degrees of their correlations were weak. Moreover, the effects of age on these parameters such as serum FGF-23, Pi, or UACR were not precisely evaluated because of the difficulties associated with age matching between pre- and postmenopausal women. Another limitation is that the present cross-sectional study was limited to Japanese females, and, thus, it remains unclear whether the results obtained may be extended to other ethnicities. Additionally, it was not possible to assess causality from a cross-sectional study. Therefore, a cohort survey on disease occurrence to evaluate direct causality among the parameters of the Pi load and its clinical effects on the kidneys and/or vasculature is necessary. The fourth limitation is that we did not obtain precise data on renal or vascular damage from the present study. Therefore, in order to demonstrate a direct relationship between Pi released into the systemic blood circulation and concomitant renal/vascular damage, a longitudinal follow-up survey on the occurrence of renal or vascular diseases in postmenopausal non-CKD women is needed.

The strength of the present study is the restricted enrollment of subjects analyzed to apparent non-CKD subjects. Under this condition, we demonstrated a negative correlation between eGFR and serum FGF-23, even when eGFR was restricted to ≥ 60 mL/min/1.73 m^2^. A novel result of the present study is that serum BAP and FGF-23, but not serum Pi, were associated with UACR in post- and premenopausal women, respectively, clearly indicating the impact of enhanced bone resorption as a source of Pi in postmenopausal women. Therefore, these results appear to support previous findings showing that the inhibition of bone resorption by anti-osteoporotic agents in postmenopausal women may protect against the development of renal damage and vascular injury, even under non-CKD conditions.

In summary, this is the first study to demonstrate the significant effects of the Pi load on UACR, a marker for renal damage and cardiovascular risk in a large number of Japanese non-CKD patients. In postmenopausal women, bone appeared to be the main source of Pi in the circulation, which caused renal and vascular damage, whereas FGF-23 by itself or as a surrogate marker of the Pi load provides a clinically useful marker of renal and vascular damage in premenopausal women.
